# Crystallization and preliminary X-ray crystallographic analysis of the inhibitory domain of the tomato mosaic virus resistance protein Tm-1

**DOI:** 10.1107/S1744309113030819

**Published:** 2013-11-29

**Authors:** Masahiko Kato, Yuichiro Kezuka, Chihoko Kobayashi, Kazuhiro Ishibashi, Takamasa Nonaka, Masayuki Ishikawa, Estuko Katoh

**Affiliations:** a Biomolecular Research Unit, National Institute of Agrobiological Sciences, Tsukuba, Ibaraki 305-0856, Japan; b Department of Structural Biology, School of Pharmacy, Iwate Medical University, Yahaba, Iwate 028-3694, Japan; c Plant–Microbe Interactions Research Unit, National Institute of Agrobiological Sciences, Tsukuba, Ibaraki 305-8602, Japan

**Keywords:** Tm-1, *Tomato mosaic virus*, replication protein

## Abstract

Tm-1, an inhibitor protein of *Tomato mosaic virus* RNA replication, contains two conserved domains: an uncharacterized domain at its N-terminus and a TIM-barrel-like domain at its C-terminus. The N-terminal domain of Tm-1 has an inhibitory activity and its three-dimensional structure has not been determined. Here, the crystallization and preliminary X-ray diffraction of the N-­terminal domain of Tm-1 are reported. A three-wavelength MAD data set was collected from a selenomethionine-labelled crystal and processed to 2.7 Å resolution. The crystal belonged to the triclinic space group *P*1, with unit-cell parameters *a* = 77.97, *b* = 105.28, *c* = 110.62 Å, α = 94.6, β = 109.3, γ = 108.0°.

## Abstract 2

A crystal of Tm-1, which is an inhibitor protein of *Tomato mosaic virus* RNA replication, was obtained using the hanging-drop vapour-diffusion method at 293 K and diffracted X-rays to 2.7 Å resolution. It belonged to space group *P*1, with unit-cell parameters *a* = 77.97, *b* = 105.28, *c* = 110.62 Å, α = 94.6, β = 109.3, γ = 108.0°.

## 1. Introduction

The tomato (*Solanum lycopersicum* L.) protein Tm-1 confers resistance against *Tomato mosaic virus* (ToMV). Tm-1 binds to ToMV replication proteins and inhibits ToMV RNA replication (Ishibashi *et al.*, 2007 ▶). The Tm-1 protein is composed of 754 amino acids and is predicted to contain two domains: an uncharacterized N-terminal domain (residues 1–431) and a C-terminal TIM-barrel-like domain (residues 484–754) (Ishibashi *et al.*, 2007 ▶). A small region (residues 79–112) within its N-terminal domain has been shown to be under positive selection during coevolution with ToMV (Ishibashi *et al.*, 2012 ▶). The purified N-terminal domain of Tm-1 expressed in *Escherichia coli* shows inhibitory activity towards ToMV RNA replication *in vitro* (Kato *et al.*, 2013 ▶). Although Tm-1 homologues are found in most plants, fungi, archaea and bacteria, the three-dimensional structure of a corresponding conserved N-terminal domain has not yet been determined.

ToMV is a positive-strand RNA virus and its genome encodes four proteins of ∼130 kDa (130K), 180 kDa (180K), 30 kDa (movement protein) and 17 kDa (coat protein) (Ishikawa & Okada, 2004 ▶). The movement protein and coat protein are dispensable for viral RNA replication, whereas the 130K and 180K (a read-through product of the 130K protein) proteins are involved in RNA replication and thus are called the replication proteins (Buck, 1999 ▶; Osman & Buck, 2003 ▶; Ishikawa *et al.*, 1986 ▶). When ToMV RNA enters a host cell, the replication proteins are translated from the genome RNA. The replication proteins associate with the genomic RNA co-trans­lationally to form a pre-membrane-targeting complex (PMTC; Komoda *et al.*, 2007 ▶). The PMTC then binds to intracellular membranes to form the replication complex, in which the negative RNA strand is synthesized using the genomic RNA as the template. Finally, the replication proteins synthesize the genomic positive RNA strand in the replication complex using the negative RNA strand as the template. Tm-1 binds the replication proteins in the PMTC and inhibits the formation of the replication complex on membranes (Ishibashi & Ishikawa, 2013 ▶).

In order to establish the structural basis of this inhibition, we have performed crystallization and preliminary crystallographic studies of the N-terminal domain fragment of Tm-1 [residues 1–431: referred to here as Tm-1(431)].

## 2. Materials and methods

### 2.1. Expression and purification of Tm-1(431) and selenomethionine-labelled Tm-1(431) [SeMet-(Tm-1(431)]

*E. coli* Rosetta(DE3) cells were transformed with the expression vector pDEST-mal-Tm-1(431) (Kato *et al.*, 2013 ▶) and cultured in Luria–Bertani medium containing 50 µg ml^−1^ ampicillin and 34 µg ml^−1^ chloramphenicol to an *A*_600_ of ∼0.5. Expression was induced by the addition of isopropyl β-d-1-thiogalactopyranoside to a final concentration of 10 µ*M*. The cells were cultured for an additional 16 h at 303 K, harvested by centrifugation at 5000*g* for 15 min and disrupted in sonication buffer (50 m*M* Tris–HCl pH 8.0, 500 m*M* NaCl, 2 m*M* β-mercaptoethanol). The lysate was clarified by centrifugation at 27 000*g* for 30 min at 277 K. The cleared lysate containing Tm-1(431) fused C-terminally to maltose-binding protein (MBP) was applied onto an MBPTrap column (GE Healthcare Bio-Sciences). The bound proteins were eluted in the aforementioned buffer containing 20 m*M* maltose and were then applied onto a HiLoad 26/60 Superdex 200 pg column (GE Healthcare Bio-Sciences) and eluted with buffer *A* (20 m*M* Tris–HCl pH 8.0, 500 m*M* NaCl, 2 m*M* β-­mercaptoethanol). Fractions containing the fusion protein were collected and again applied onto the MBPTrap column to concentrate the protein. The fusion protein was then cleaved with factor Xa (Novagen) by incubation at 293 K for 16 h. Tm-1(431) was separated from the MBP and uncleaved fusion protein by chromatography through loading onto a HiLoad 26/60 Superdex 75 pg column (GE Healthcare Bio-Sciences) equilibrated with buffer *B* (20 m*M* Tris–HCl pH 8.0, 150 m*M* NaCl, 1 m*M* dithiothreitol). The Tm-1(431)-containing fractions were applied onto the MBPTrap column to remove any remaining MBP and the recovered flowthrough fraction was applied onto a HiLoad Q HP column (GE Healthcare Bio-Sciences). Bound Tm-1(431) was eluted with a 150–500 m*M* NaCl linear gradient in buffer *B* and Tm-1(431)-containing fractions were applied onto a HiLoad 26/60 Superdex 75 pg column (GE Healthcare Bio-Sciences) equilibrated with buffer *B*. The Tm-1(431)-containing fractions were dialyzed overnight against dialysis buffer (20 m*M* Tris–HCl pH 8.0, 50 m*M* NaCl, 1 m*M* dithiothreitol) and concentrated to 10 mg ml^−1^ in an Amicon Ultra-4 centrifugal filter unit (Millipore). Additional details of the expression and purification of Tm-­1(431) have been reported in Kato *et al.* (2013 ▶).

To express SeMet-Tm-1(431), *E. coli* B834(DE3)pRARE cells were transformed with the expression vector pDEST-mal-Tm-1(431). The expression of SeMet-Tm-1(431) in the B834(DE3)pRARE cells and the purification of the protein were performed as described above except that LeMaster medium (LeMaster & Richards, 1985 ▶) was used as the culture medium.

### 2.2. Crystallization and X-ray data collection

Native Tm-1(431) and SeMet-Tm-1(431) were crystallized using the hanging-drop vapour-diffusion method at 293 K. The crystallization drops were prepared on a siliconized cover slip by equilibrating a mixture consisting of 1.5 µl protein solution (10.0 mg ml^−1^ protein in 20 m*M* Tris–HCl pH 8.0, 50 m*M* NaCl, 1 m*M* dithiothreitol) and 1.5 µl reservoir solution against 400 µl reservoir solution.

To improve the diffraction quality, we assessed the effect of various post-crystallization treatments (Heras & Martin, 2005 ▶) using the native crystal as the subject (see §[Sec sec3]3). After crystal growth, the chosen post-crystallization treatment involved stepwise equilibration of the native and SeMet-labelled crystals against reservoirs containing increasing concentrations of ethylene glycol [EG; 2.5, 5, 12.5 and 25%(*w*/*v*)] with incubation intervals of 6–12 h at 293 K. The crystals were then flash-cooled in a cryogenic stream of nitrogen gas without the addition of a cryoprotectant.

A three-wavelength MAD data set was collected from an SeMet-Tm-1(431) crystal on the BL38B1 beamline at SPring-8 at 100 K. The MAD experiment wavelengths were determined by an XAFS experiment on an SeMet-labelled crystal. The crystal-to-detector distance was set to 300 mm and the diffraction images were recorded using a Quantum 315 CCD detector (Area Detector Systems Corporation) with a 1° oscillation and 5 s (peak and edge data) or 4 s (remote data) exposure per frame. A total of 720 frames were collected with an oscillation range of 720° for the peak and edge data. A total of 180 frames were collected with an oscillation range of 180° for the remote data.

The collected data set was processed using *MOSFLM* (Powell, 1999 ▶) and *SCALA* (Evans, 2006 ▶) as implemented in *xia*2 (Winter, 2010 ▶). An anomalous difference Patterson map for the peak data set was calculated using *FFT* (Read & Schierbeek, 1988 ▶).

## 3. Results and discussion

**Figure 1:**
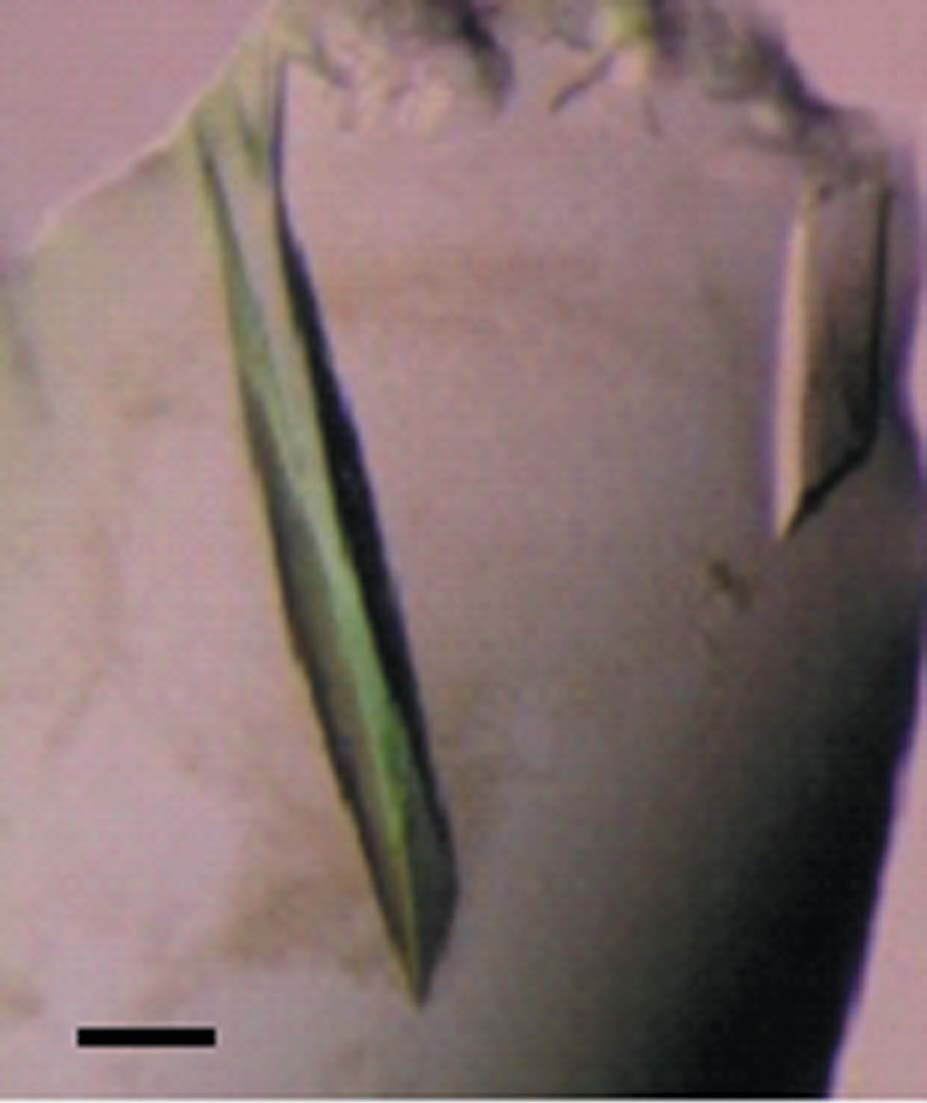
Tm-1(431) crystals. The typical crystal dimensions are approximately 0.6 × 0.3 × 0.07 mm. The scale bar indicates 0.1 mm.

The initial crystallization screening for native Tm-1(431) was performed using reagents from commercially available screening kits (Wizard I, II and III from Emerald BioSystems and Crystal Screen, Crystal Screen 2 and PEG/Ion from Hampton Research). In the first screening, small crystals (0.02–0.05 mm in the longest dimension) were observed for many of the conditions in PEG/Ion [*e.g.* 20%(*w*/*v*) PEG 3350 and 0.2 *M* salt]. Relatively large crystals were observed when the reservoir solution contained an ammonium or a potassium salt. To optimize the crystallization conditions, reservoir solutions with combinations of PEGs with various average molecular weights (*e.g.* 3350, 4000, 6000 and 8000) and several ammonium (*e.g.* NH_4_Cl, NH_4_NO_3_) or potassium salts (*e.g.* KCl, Na,K tartrate) were tested. The combination of ammonium tartrate dibasic and PEG 8000 produced three-dimensional crystals, whereas the other conditions produced thin crystals which diffracted anisotropically in preliminary in-house experiments. The most promising conditions for the reservoir solution were found to be 0.4 *M* ammonium tartrate dibasic, 7.5%(*w*/*v*) PEG 8000. Native Tm-1(431) crystals grew within about 7 d under these conditions to approximate dimensions of 0.6 × 0.3 × 0.05 mm (Fig. 1 ▶).

**Figure 2:**
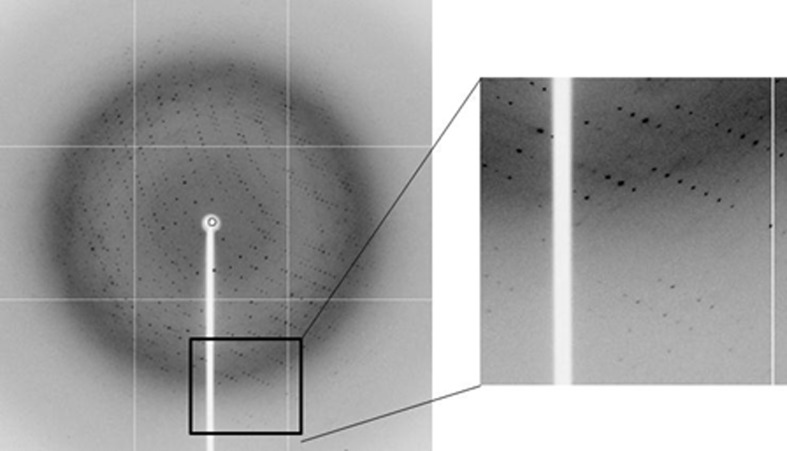
X-ray diffraction pattern of an SeMet-Tm-1(431) crystal, which diffracted to beyond 2.7 Å resolution.

To assess the ability of native Tm-1(431) crystals grown without post-treatment to diffract X-rays, they were transferred into and soaked briefly in 20%(*v*/*v*) EG and then flash-cooled in a cryogenic stream of nitrogen gas. However, crystals cryoprotected in this manner diffracted X-rays poorly to only 4–5 Å resolution. To improve the diffraction quality, we tested post-crystallization treatment (Heras & Martin, 2005 ▶). After crystal growth, the crystallization drop was serially equilibrated against reservoirs containing increasing concentrations of EG [2.5, 5, 12.5 and 25%(*w*/*v*)] with incubation times of 6–12 h. This serial equilibration promoted further vapour diffusion of the crystallization drop and led to dehydration of the crystals. The dehydrated crystals exhibited a slight (1.0%) reduction in unit-cell volume compared with the nondehydrated crystals (data not shown). Dehydration might have caused the protein molecules to pack more closely together, consequently yielding improved diffraction. However, dehydration would have also concentrated the crystallization solutes, *e.g.* PEG 8000, in the crystallization drop. The concentrated PEG environment probably enabled us to avoid soaking the crystals in 20%(*v*/*v*) ethylene glycol as a means of cryoprotection, which might have caused the poor diffraction. The crystals finally diffracted to beyond 2.7 Å resolution (Fig. 2 ▶) owing to the post-crystallization treatment.

**Figure 3:**
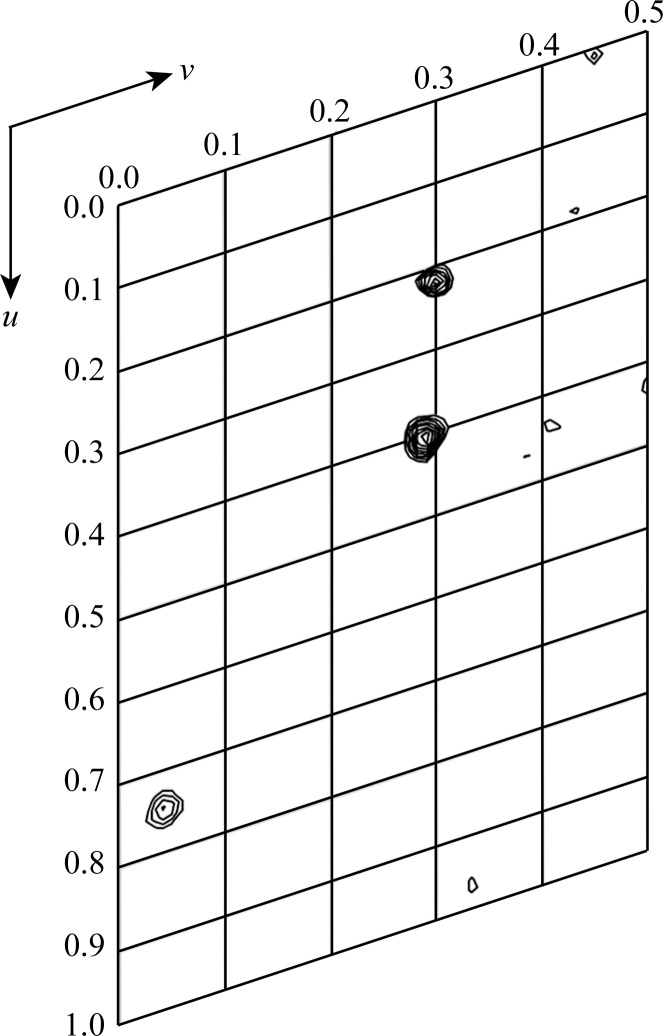
A section at *w* = 0.16 of the anomalous difference Patterson map for the SeMet-Tm-1(431) crystal. The map was calculated using the peak data set and is contoured at intervals of 0.5σ starting at 2.5σ above the mean density level.

SeMet-Tm-1(431) was crystallized in the same manner as native Tm-1(431) and the crystal had almost the same quality as the native crystals. X-ray diffraction data were obtained from an SeMet-Tm-­1(431) crystal on beamline BL38B1 at the SPring-8 synchrotron-radiation source, Harima, Japan after post-crystallization treatment. A three-wavelength MAD data set was collected from the SeMet-labelled crystal at 100 K. The crystal belonged to the triclinic space group *P*1, with unit-cell parameters *a* = 77.97, *b* = 105.28, *c* = 110.62 Å, α = 94.6, β = 109.3, γ = 108.0°. Assuming six Tm-1(431) monomers per asymmetric unit, the Matthews coefficient *V*_M_ is 2.9 Å^3^ Da^−1^, which is a reasonable value and corresponds to a solvent content of 57% (Matthews, 1968 ▶). Data-collection and processing statistics are summarized in Table 1 ▶. The peak data set (λ = 0.9788 Å) showed strong peaks derived from the Se atoms in the anomalous difference Patterson map contoured at 2.5σ above the mean density level (Fig. 3 ▶). MAD phasing, density modification and initial model building using *AutoSol* from the *PHENIX* program suite (Adams *et al.*, 2010 ▶) are now in progress.

## Figures and Tables

**Table 1:** Data-collection and processing statistics for SeMet-Tm-1(431) Values in parentheses are for the highest resolution shell.

	Peak	Edge	Remote
Experimental conditions			
Beamline	BL38B1, SPring-8		
Wavelength (Å)	0.9788	0.9792	0.9639
Temperature (K)	100		
Detector	ADSC Quantum 315		
Oscillation angle (°)	1.0	1.0	1.0
Exposure time (s)	5.0	5.0	4.0
No. of images	720	720	180
Intensity statistics			
Space group	*P*1		
Unit-cell parameters (Å, °)	*a* = 77.97, *b* = 105.28, *c* = 110.62, α = 94.6, β = 109.3, γ = 108.0		
Software used	*MOSFLM*/*SCALA*		
Resolution range (Å)	49.54–2.89 (3.04–2.89)	49.52–2.90 (3.06–2.90)	49.63–2.71 (2.86–2.71)
*R*_merge_ † (%)	4.9 (24.5)	4.7 (23.1)	5.7 (23.7)
Completeness (%)	90.4 (80.4)	80.1 (74.7)	95.0 (95.5)
〈*I*/σ(*I*)〉	12.3 (3.1)	12.8 (3.3)	10.2 (3.1)
No. of observed reflections	482521 (63400)	423404 (58192)	155953 (23027)
No. of unique reflections	62196 (8104)	54549 (7433)	79784 (11683)
Multiplicity	7.8 (7.8)	7.8 (7.8)	2.0 (2.0)

† *R*_merge_ = 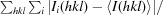 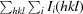, where *I_i_*(*hkl*) is the intensity of the *i*th observation and 〈*I*(*hkl*)〉 is the mean intensity of the reflections.
